# De Novo Transcriptome Sequencing and the Hypothetical Cold Response Mode of *Saussurea involucrata* in Extreme Cold Environments

**DOI:** 10.3390/ijms18061155

**Published:** 2017-06-07

**Authors:** Jin Li, Hailiang Liu, Wenwen Xia, Jianqiang Mu, Yujie Feng, Ruina Liu, Panyao Yan, Aiying Wang, Zhongping Lin, Yong Guo, Jianbo Zhu, Xianfeng Chen

**Affiliations:** 1 College of Life Sciences, Shihezi University, Shihezi 832000, China; lj6966324@126.com (J.L.); xiashz@126.com (W.X.); jianqiangmshz@163.com (J.M.); fengyujie@sina.com (Y.F.); shuilingzi123_4@126.com (R.L.); way-sh@126.com (A.W.); 2 Department of Regenerative Medicine, Tongji University School of Medicine, Shanghai 200065, China; hailiang_1111@tongji.edu.cn; 3 ShengTing Bioinformatics Institute, Christiansburg, VA 24073, USA; yolanda_yan@shengtinggroup.com; 4 College of Life Sciences, Perking University, Beijing 100871, China; linzp@pku.edu.cn; 5 Institute of Crop Science, Chinese Academy of Agricultural Sciences, Beijing 100081, China; guoyong@caas.cn

**Keywords:** *Saussurea involucrata*, RNA-Seq, transcriptome, signal transduction, transcription factor, free radical

## Abstract

*Saussurea involucrata* grows in high mountain areas covered by snow throughout the year. The temperature of this habitat can change drastically in one day. To gain a better understanding of the cold response signaling pathways and molecular metabolic reactions involved in cold stress tolerance, genome-wide transcriptional analyses were performed using RNA-Seq technologies. A total of 199,758 transcripts were assembled, producing 138,540 unigenes with 46.8 Gb clean data. Overall, 184,416 (92.32%) transcripts were successfully annotated. The 365 transcription factors identified (292 unigenes) belonged to 49 transcription factor families associated with cold stress responses. A total of 343 transcripts on the signal transduction (132 upregulated and 212 downregulated in at least any one of the conditions) were strongly affected by cold temperature, such as the *CBL-interacting serine/threonine-protein kinase* (*CIPKs*), *receptor-like protein kinases*, and *protein kinases*. The circadian rhythm pathway was activated by cold adaptation, which was necessary to endure the severe temperature changes within a day. There were 346 differentially expressed genes (DEGs) related to transport, of which 138 were upregulated and 22 were downregulated in at least any one of the conditions. Under cold stress conditions, transcriptional regulation, molecular transport, and signal transduction were involved in the adaptation to low temperature in *S. involucrata*. These findings contribute to our understanding of the adaptation of plants to harsh environments and the survival traits of *S. involucrata*. In addition, the present study provides insight into the molecular mechanisms of chilling and freezing tolerance.

## 1. Introduction

Temperature is an important environmental factor that affects crop production and quality in agriculture. A deteriorating environment characterized by extremely low temperatures diminishes crop productivity, which is a problem for the food supply of a continuously growing population. The response to cold temperature in plants is a complex process that involves morphological, physiological, biochemical, and molecular processes [1,2,3,4,5]. Plants are divided into two types according to their response to cold, namely cold sensitive and cold tolerant plants. Plants that possess the ability to endure cold stress originate mainly from temperate areas, whereas chilling and freezing stresses are typically experienced by plants from tropical or sub-tropical zones. Certain sub-tropical plants that are placed under cold conditions for 7 days increase their freezing tolerance capabilities in a process termed cold acclimation [6]. Evidence suggests that transcription factors (TFs), plant hormones, noncoding RNAs, protein modifiers, and epigenetic modifications are involved in the complex network of the low temperature response [7,8,9,10].

*Saussurea involucrata* Kar. et Kir., which only flowers once during its life cycle, is a perennial species of the Asteraceae and *Saussurea genus*. The herbs grow in high mountain areas at approximately 2400–4100 meters and are covered by snow throughout the year. The plants experience extreme temperature changes within a day from summer to winter. Under these conditions, natural selection in *S. involucrata* has led to the development of stable physiological and biochemical mechanisms to endure environmental stresses such as extreme temperatures, intense radiation, and hypoxia. The habitat of *S. involucrata*, which is characterized by severe cold and high radiation throughout the year, make it an important species to study cold tolerance in plants. Some genes related to cold tolerance in *S. involucrata* have been cloned and transformed into *Arabidopsis*, *tobacco*, and *Solanum lycopersicum*, resulting in increased stress tolerance in these plants [11,12].

Next generation sequencing (NGS) technologies allow genome-wide expression profiling of non-model species [13]. The genomes of several species are available or were previously explored by re-sequencing and de novo sequencing [14,15]. The sequencing data is useful for investigating genes from coding RNA to non-coding RNA to analyze the transcription expression profile. NGS has been used to identify pathways involved in the regulation of genes associated with growth and development or stress tolerance under various conditions. The species investigated include plants such as *Arabidopsis* [16], *cotton* [17], *M. tuncatula* [18,19,20,21,22] and *Brassica napus* [23]. Software has been developed for the de novo assembly, annotation, and statistical analyses of expression levels [24,25,26,27,28], making transcriptome analysis possible.

Although some genes and pathways related to cold stress have been identified, most studies are based on plants that do not grow in cold temperatures during their entire lifetime. The aim of the present study was to gain a general understanding of how *S. involucrata* senses and resists cold stress. Six different temperatures and times were selected to sequence the transcriptome, and the cold responsive genes of *S. involucrata* were analyzed using NGS technologies. The integral expression profile of whole genome regulation under different temperatures and times was analyzed. In addition, a number of genes involved in pathways were analyzed, such as signal transduction, membrane protein, transport, and transcriptional regulation.

## 2. Results and Discussion

### 2.1. Transcriptome Sequencing, Assembly, and Annotation

**Figure 1:**
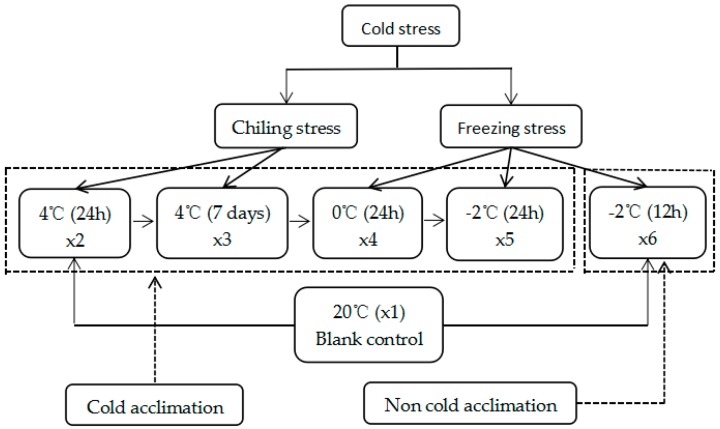
The process of the cold stress on the *S. involucrate* seedlings.

There are differences between cold acclimation and the lack of cold acclimation in plants. In general, the ability for cold tolerance would be enhanced after cold acclimation, or the experience of 7 days of 4 °C treatment. Therefore, the conditions of 4 °C (24 h) (×2), 4 °C (7 days) (×3) were selected for cold treatment. Moreover, the responsive mechanism is different between chilling and freezing temperatures in plants. Accordingly, 20 °C → 4 °C (7 days) → 0 °C (24 h) (×4), 20 °C → 4 °C (7 days) → 0 °C (24 h) → −2 °C (24 h) (×5), and directly at −2 °C for 12 h from 20 °C (×6) were selected as the freezing treatment conditions (Figure 1).

Total RNA was extracted from the seedlings of *S. involucrata* treated with different cold stresses and sequenced by the Illumina HiSeq 2500 platform. A total of 46.8 Gb clean data (233,738,293 clean reads) were acquired after removing adaptor sequences, ambiguous nucleotides, and low quality sequences (Table 1). A total of 199,758 transcripts in the range of 201–15,818 base pairs (bp) with a N50 length of 1099 bp were produced using the software Trinity [29] (Figure S1). The average and median sequence lengths were 711.37 and 421.00 bp, respectively.

Moreover, 60,501 peptides were produced by translating all of the unigenes. Annotation of these peptides showed that 57,046 peptide sequences (94.29%) had significant matches with the NCBI NR.aa dataset and 41,002 (67.77%) peptide sequences had significant matches in the EBI SwissProt. In total, 92.3% (184,416) of the transcripts were successfully annotated in at least one of the databases of the NCBI (NR.aa, EBI SwissProt, Pfam, EggNOG, gene ontology (GO), and clusters of orthologous groups (COG)) (Figure S2; Table S1).

To further evaluate the integrity of the transcriptome library and classify gene functions, the unigenes were searched against the COG database. A total of 71,165 unigenes were assigned to 23 COG categories (Figure S3). Among these categories, the cluster for signal transduction mechanisms represented the largest group (3250), followed by general function prediction only (2921), function unknown (1911), post-translational modification, protein turnover, chaperones (1792), replication, and recombination and repair (1668). Only a few unigenes were assigned to the category of cell motility.

### 2.2. Differential Expression Analysis of the Assembled S. involucrata Transcripts under Different Chilling Temperatures

**Figure 2:**
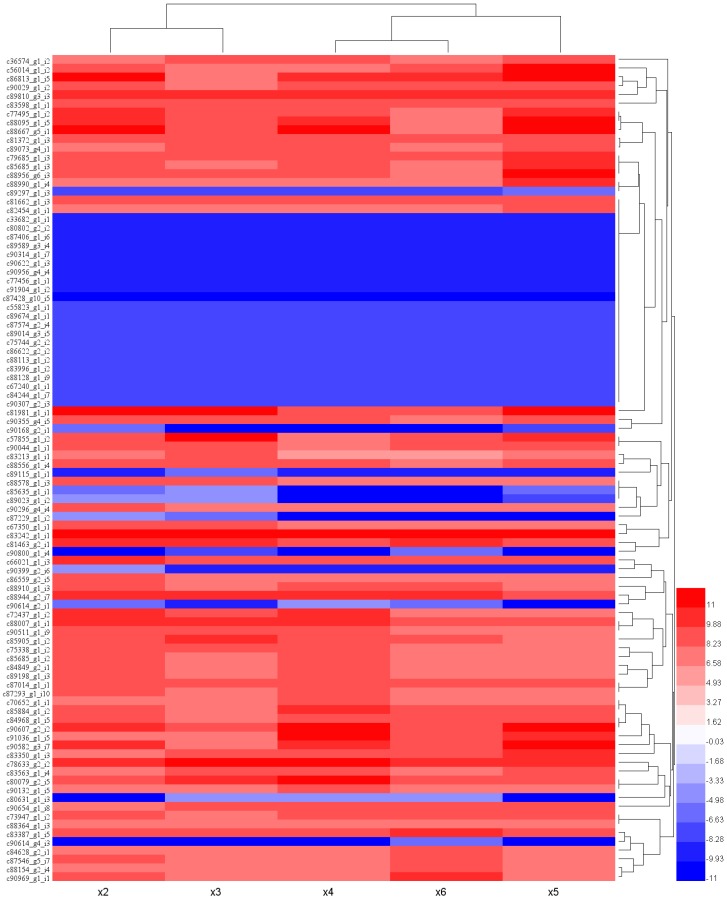
Heat map of the expression profiles with |logFC| ≥ 2 in all cold treatments. Columns and rows in the heat maps represent samples and unigenes, respectively. Sample names are displayed below the heat map. The color scale indicates fold changes of gene expression.

Differential expression analysis was performed between the ×1 (20 °C) and different cold stresses. Differentially expressed genes (DEGs) (BCV = 0.2, logFC ≥ 2 and FDR < 0.001) were defined (Figure S4). A total of 28 (1 upregulated and 27 downregulated), 105 (2 upregulated and 103 downregulated), 179 (154 upregulated and 25 downregulated), 1145 (448 upregulated and 697 downregulated) and 48 (14 upregulated and 34 downregulated) DEGs were identified in the different cold treatments (×2, ×3, ×4, ×5, and ×6, respectively; Table S2). Clustering of these DEGs with |logFC| ≥ 2 in all five conditions showed that 94 transcripts were in the cluster (Figure 2). In these genes, 66 transcripts were novel and 9 transcripts were reported previously in response to cold stress. These genes related to signal transduction, protein transport, transcription, DNA repair, chromosome modification, and other functions.

### 2.3. Cluster of Orthologous Groups of Proteins Classification of the Differentially Expressed Genes

**Figure 3:**
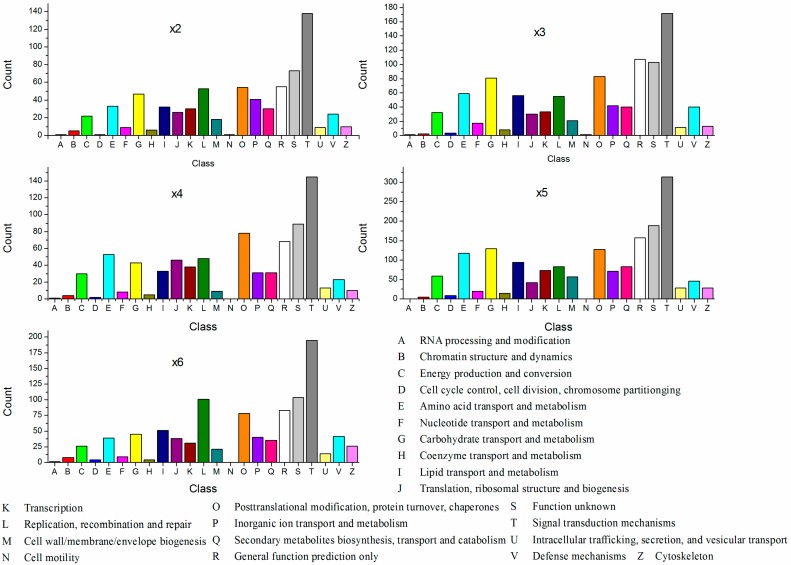
Clusters of orthologous groups (COG) classification of the differentially expressed genes of the samples under different cold temperatures.

To elucidate the functional classification of the DEGs, the categories were searched against the COG database. The results showed that the common categories of “signal transduction”, “general function prediction only” and “function unknown” were the most enriched in all samples (Figure 3). However, some categories were different among the different samples. The differences were mainly in gene numbers and categories. For example, in the comparison of ×2 and ×3, the categories of nucleotide transport and metabolism had different numbers of unigenes. Based on the comparison between the samples, the category of cell motility only emerged at 4 °C. The term of RNA processing and modification was not emerged in the ×4 and ×5. By the statistic, the transcript number of the category of Signal transduction mechanism was 138, 172, 145, 314, and 195 in the ×2, ×3, ×4, ×4, ×5, and ×4 respectively. The trend in the transcript number of the category of Inorganic ion transport and metabolism and Lipid transport and metabolism resembled the category of Signal transduction mechanism.

### 2.4. The Transcriptional Regulatory Network Is Involved in the Adaptation of S. involucrata to Extreme Cold Environments

**Figure 4:**
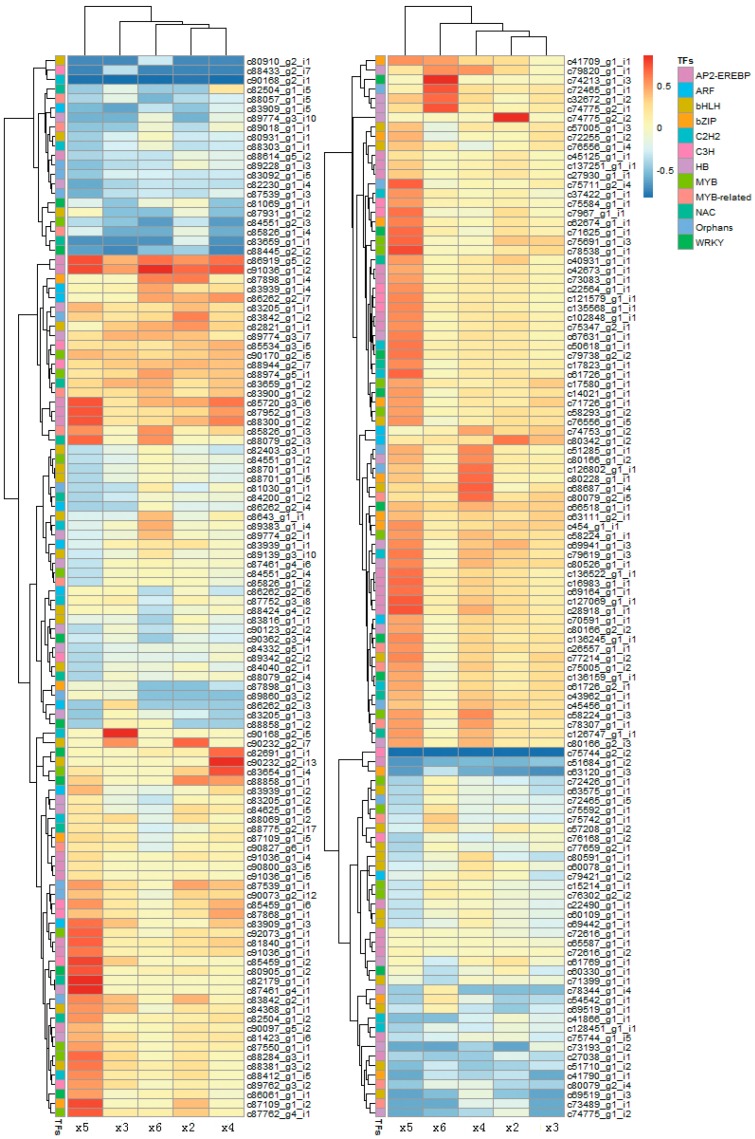
Analysis of the expression levels of the 12 top transcription factor families. Columns and rows in the heat maps represent samples and transcript-ID, respectively. Sample names are displayed below the heat map. Color scale indicates fold changes of gene expression. Family names are represented by the color map on the top right corner of the figure.

**Figure 5:**
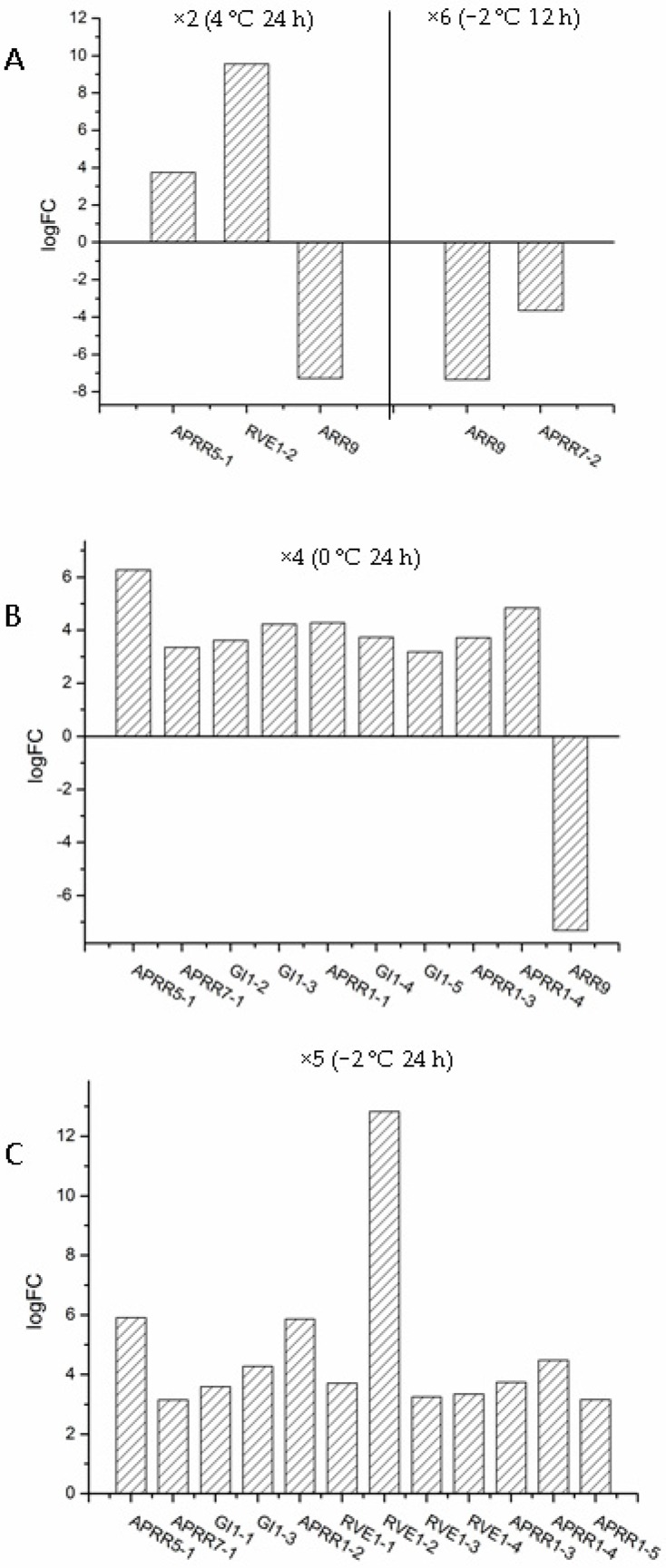
The expression level of the unigenes involved in the circadian rhythms. The logFC value of the differentially expressed unigene was used for the bar graph. The A, B and C represent the different temperature and time conditions: (**A**) 4 °C (24 h) and −2 °C (12 h); (**B**) 0 °C (24 h); **(C**) −2 °C (24 h).

The DEGs were searched by analysis of both Pfam (Available online: http://www.sanger.ac.uk/Software/Pfam/) and PlnTFDB (Available online: http://plntfdb.bio.uni-potsdam.de/v3.0/blastform.php). In total, 301 TFs (unigenes that comprise 365 transcripts) belonging to 49 TF families showed significant changes in expression level under cold conditions (Supplemental Figure S5). The 12 top TF families were AP2-EREBP, HB, bHLH, ARF, MYB-related, Orphans, MYB, NAC, bZIP and WRKY (Figure 4). Proteins belonging to the AP2-EREBP family are essential for the transcriptional regulation of a variety of biological processes related to growth, development and abiotic stress tolerance. In *S. involucrata*, 24 transcripts that the proteins belonged to the AP2-EREBP family were upregulated and 3 transcripts were downregulated under all cold stress conditions. There were two special factors that were only upregulated at ×4 and ×5, which are *Floral homeotic protein APETALA 2* (*AP2*) and *Ethylene-responsive transcription factor RAP2-7* (*RAP2-7*) respectively. The AP2 associating with the specification of floral identity is related to heat, salt and drought stresses. The RAP2-7 linked to circadian clock can bind the GCC box (a *cis*-acting element) to regulate gene expression involved in the cold stress. The WRKY family plays significant roles in the responses to abiotic and biotic stresses. In the WRKY family, two unigenes (*Probable WRKY transcription factor 19*; *Probable WRKY transcription factor 17*) were upregulated and two (*Probable WRKY transcription factor 70*; *Probable WRKY transcription factor 26*) were downregulated under all cold stresses. The transcripts, which the protein belonged to the Pseudo ARR-B family, were always upregulated or downregulated in at least one of the cold conditions (*Two-component response regulator-like APRR5*, *Two-component response regulator-like APRR1*, *Two-component response regulator ARR2* etc.), suggesting that the circadian rhythm-plant pathway was important for the ability of *S. involucrata* to endure temperature changes within the day from summer to winter (Figure 5).

Similar to *Arabidopsis*, the genes related to the protein belonged to the AP2/EREBP superfamily, NAC family, and WRKY family, and were upregulated under all cold stresses [30,31]. The TF families of DRE1, WRKY, NAC, bZIP, MYB, and MYC are necessary for cold acclimation [32]. Among these TFs, the mechanism of the DRE1 subfamily (include *DRE1A*/*CBF3*, *DRE1B*/*CBF1* and *DRE1C*/*CBF2*) was clearer than that of the others, and it regulates other genes such as Late Embryogenesis Abundant Proteins/LEA proteins (termed the CBF regulon), which function together to enhance the freezing tolerance of *Arabidopsis* [33,34]. The *Solanum lycopersicum* has a functional factor similar to *AtCBF3*, but its regulon differs from that of *Arabidopsis* [35]. In *S. involucrata*, as a cold tolerant plant, *DRE1A* and *DRE1B* were upregulated under cold stress and showed a peak at −2 °C for 24 h (×5). However, *DRE1C*, a known suppression factor of *DRE1A*, which regulates the other pathways related to cold acclimation [36,37,38], was not found in the transcriptome.

In comparison with *Arabidopsis*, some genes of TF families were differentially expressed in *S. involucrata* under cold stresses, such as FHA, PHD, RWP-RK, ABI3VP1, AUX/IAA, TCP, SBP, SWI/SNF-BAF60b, Sigma70-like, LUG, DDT, E2F-DP, Jumonji, BSD, Coactivator p15, and CPP. The genes of the FHA family function in DNA repair and signal transduction [39,40]. The genes of the AUX/IAA family, which play a role in repressing the expression of primary/early auxin responsive genes [41,42,43], affect the expression of auxin response factors. Genes in the sigma70-like family regulate plastid gene expression [44,45,46]. The chloroplast is important for cold acclimation and freezing tolerance, and functions as a sensor in the response of plants to cold and light [47]. Under cold stress conditions, chromatin modification is important for the cold and freezing tolerance of plants [48].

### 2.5. Cold Regulation of Signal Transduction Components

There were 343 transcripts that showed up or downregulation in at least any one of five conditions involved in cold adaptation about the signal transduction in all samples. Based on the protein characteristics, the transcripts were assigned to eight categories, such as protein kinase, protein phosphatase, receptor-like kinase, histidine kinase, rhythm signal, GTP-related, lipid signal, and calcium-related (Table 2). Among these, there were 40 and 52 transcripts upregulated and downregulated in ×2 (4 °C (24 h)), respectively; 44 and 55 transcripts upregulated and downregulated in ×3 (4 °C (7 days)); 61 and 49 transcripts upregulated and downregulated in ×4 (0 °C (24 h)); 120 and 148 transcripts upregulated and downregulated in ×5 (−2 °C (24 h)); 60 and 106 transcripts upregulated and downregulated in ×6 (−2 °C (24 h)). The receptor-like kinase represented the largest group among the DEGs in comparison with the other categories. In plants, the mechanism of signal transduction is different under cold and freezing stresses [49]. Moreover, freezing adaptation involves the regulation of more genes associated with signal transduction than cold adaptation in *S. involucrata* in general.

The CBL-interacting proteins of *S. involucrata* were upregulated in all samples except in ×3. However, all the calcium-dependent protein kinases were downregulated in all samples. Moreover, the other types of calcium-related proteins were upregulated, namely *the probable calcium-binding protein CML* and *the calcium-binding protein PBP*. Ca^+2^ is an essential signaling component for early cold signal transduction, and Ca^+2^ binding proteins are the main signaling molecules in the early response to cold stress. In the early time points of cold stress, two Ca^+2^ binding proteins, CBL-interacting protein kinase 9 and calcium-dependent protein kinase, regulate eight calcium related proteins [30].

In present, lipid signaling genes were not be identified in *S. involucrata*. In this study, there were 22 genes involved in lipid signaling: *phospholipase A*, *phospholipase D*, *phospholipase C*, *phospholipase SGR*, and *inositol 1,4,5-trisphosphate 5-phosphatase*. The PLCs act on phosphatidylinositol 4,5-bisphosphate, generating inositol 1,4,5-trisphosphate (IP3) and diacylglycerol (DAG). IP3 triggers Ca^+2^ releases from internal stores [50]. The *Phosphoinositide phospholipase C 7*, *Phospholipase D p1* are induced by cold in the *Arabidopsis* (Available online: https://genevisible.com/perturbations/AT/UniProt/Q9LY51; Available online: https://genevisible.com/perturbations/AT/UniProt/Q9LRZ5) and the *Phospholipase A1-II 5* is a cold responsive gene in the *Oryza* (Available online: https://genevisible.com/perturbations/OS/UniProt/Q5NAI4).

The receptor-like protein kinases were associated with the second signal generated from the early cold response. *Leucine-rich repeat transmembrane protein kinase*, *RKL1* (At1g48480) is induced by wounding, pathogens, and salicylic acid in *Arabidopsis* [51,52]. *The receptor-like protein kinase 1* (*RPK1*) gene (At1g69270) is induced by abscisic acid (ABA), dehydration, high salt, and cold [53,54]. The number of receptor-like protein kinases in *S. involucrata* under the cold stresses was more than that in *Arabidopsis*. These large numbers of proteins may be produced to signal more quickly from the extracellular to the intracellular environment under cold stress in *S. involucrata*. Meanwhile, many genes associated with circadian rhythms showed expression changes, such as *the pseudo response regulator APRR1*, *pseudo response regulator APRR5*, and *pseudo response regulator APRR9*. In *Arabidopsis*, *APRR1*, *APRR5* and *APRR9* are cold responsive genes, of which *APRR5* and *APRR9* were upregulated at 4 °C and *APRR5* and *APRR1* were upregulated at 0 °C [30,31]. In *S. involucrata*, *APRR5* was upregulated in ×2 (4 °C (24 h)), ×4 (0 °C (24 h)), and ×5 (−2 °C (24 h)), and *APRR1*, *APRR5*, *APRR7*, and *APRR9* were upregulated in ×4 (0 °C (24 h)). In addition, many other factors associated with circadian rhythms changed their expression level under cold stress, such as *REVEILLE*, *GIGANTEA* and *flavin-binding kelch repeat F-box protein 1* and the others. *FHY1* (*FAR-RED ELONGATED HYPOCOTYL 1*), which can regulate the PHYA shuttling from the cytoplasm to the nucleus, was only upregulated at the freezing temperature or lower. In the *Arabidopsis*, the circadian rhythms act as upstream control system could regulate the expression of the *DRE1A*. Wherefore, we could infer that the pathway of circadian rhythms was important in freezing tolerance, by the way of regulation of *FHY*, *APRR1*, *APRR5*, and *GIGANTEA* etc. for *S. involucrate*.

### 2.6. Membrane Proteins Associated with Transport Play Important Roles in Cold Adaptation

Membrane proteins associated with transport are important for cold adaptation. The transporters among DEGs in *S. involucrata* were involved in many aspects, such as the transport on the carbohydrates, ion-related, organic substance, inorganic salt, amino acid, and water (Table 3). The five categories showing the greatest number of genes under cold stress were ion-related, amino acid, ABC family (ATP-Binding Cassette, ABC), NRT1/PTR family, and organ-related. The category of organ-related included Niemann-Pick C proteins, Folate transporters, organic cation/carnitine transporters, CMP-sialic acid transporters, lipid-transfer proteins, phosphatidylinositol/phosphatidylcholine transfer proteins, inositol transporters, malate transporters, and phospholipid-transporting ATPases. Inositol and phosphatidylinositol transporters are involved in calcium signaling. The ion-related transporter plays a role in maintaining the normal cell morphology by regulating the osmotic pressure and protein cofactors associated with normal metabolism. The ABC family is a large transporter family involved in the transport of substances from small ions to large proteins. MDR1 and MDR3 belong to the ABC superfamily and act as a type of flippase that causes membrane phase changes by lipoid and protein rearrangement [55,56].

Comparison of *S. involucrata* with *Arabidopsis* showed that the genes belonged ABC family, nucleotide, auxin-related, and amino acid at ×2 (4 °C (24 h)); nucleotide, auxin-related, NRT1/PTR family, and amino acid at ×3 (4 °C (7 days)); and ABC super family, aquaporin, and NRT1/PTR family at ×4 (0 °C (24 h)) were responsive to cold stress in *S. involucrata* or *Arabidopsis*.

ABC family members have been mainly studied in microorganisms and humans. They play roles in ion transport, inorganic salt transport, carbohydrate transport, protein transport, and drug resistance [56]. The category NRT1/PTR family, which is involved in the transport of nitrogen, was always detected at ×2 (4 °C (24 h)), ×3 (4 °C (7 days)), and ×4 (0 °C (24 h)). The aquaporin related protein is a kind of water channel protein that is responsible for maintaining normal water metabolism. Its expression was inhibited by Ca^+2^ in *Lilium lancifolium* at 4 °C [57]. However, both upregulated and downregulated aquaporins were detected in all samples of *S. involucrata*. This suggests that there is another mechanism regulating aquaporins in *S. involucrata*.

### 2.7. Quantitative Real-Time-PCR Validation of Differentially Expressed Transcripts from RNA-Seq

**Figure 6:**
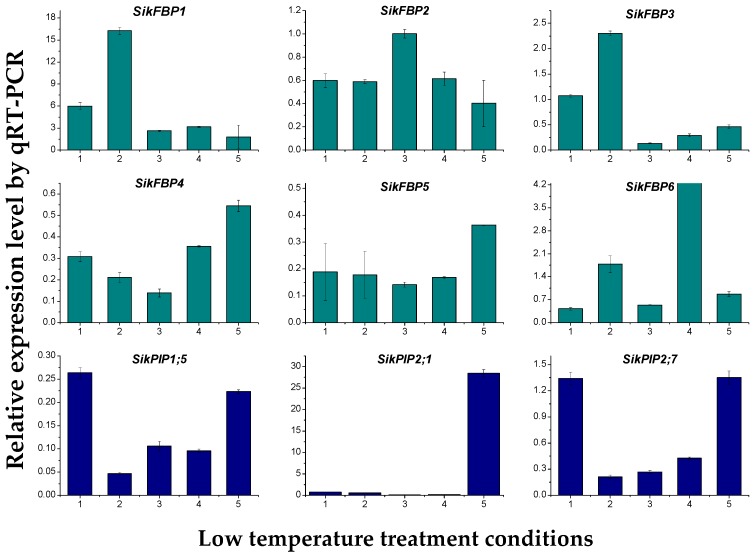
Expression analysis of nine randomly selected RNA-Seq genes from *S. involucrata* under different cold conditions. The *GAPDH* gene was used as a reference gene for normalization of gene expression data. The lateral axis (1, 2, 3, 4, and 5) represents the different cold stresses: 20 °C → 4 °C for 24 h (×2), 20 °C → 4 °C for 7 days (×3), 20 °C → 4 °C for 7 days → 0 °C for 24 h (×4), 20 °C → 4 °C for 7 days → 0 °C for 24 h → −2 °C for 24 h (×5), 20 °C → −2 °C for 12 h (×6), respectively. Different color bars represent different gene function patterns.

**Figure 7:**
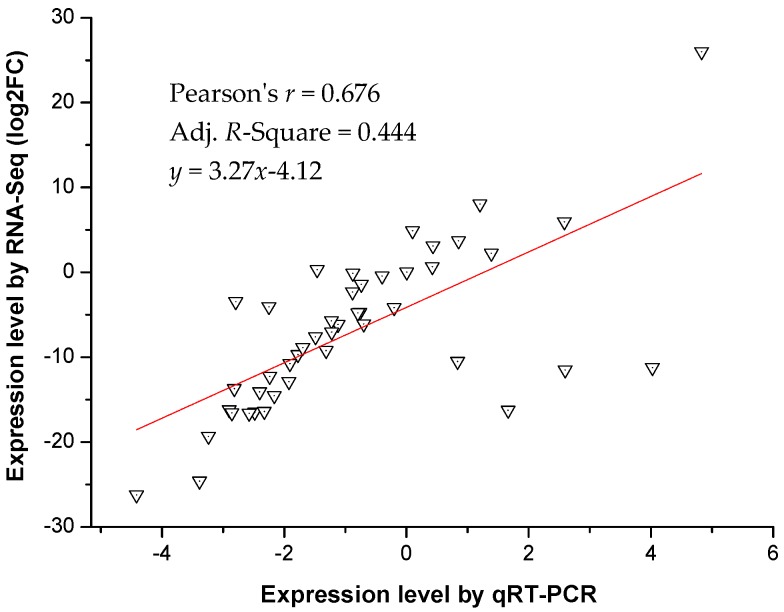
Correlations of expression levels analyzed by real time PCR (qPCR, *x* axis) with data obtained using the log2 RNA-Seq platform (*y* axis) for different temperatures and time points.

Nine randomly selected transcripts were used to confirm the expression patterns of the Illumina RNA-Seq results by quantitative real-time PCR (qRT-PCR) (Figure 6). Three genes encoding plasma membrane intrinsic proteins (*SikPIP2-1*, *SikPIP2-2*, and *SikPIP2-7*) were related to membrane transport of water, glycerinum, and small molecule solutes. Six genes encoding fructose biphosphate aldolase (*SikFBP1*, *SikFBP2*, *SikFBP3*, *SikFBP4*, *SikFBP5*, and *SikFBP6*) were involved in the pathways of glycolysis, gluconeogenesis, and carbon fixation. The qRT-PCR results showed a similar change trend for all tested transcripts at the different cold temperatures and time points (Figure 5). *SikFBP1* was upregulated when the temperature gradually decreased from 20 to −2 °C, showing the greatest change at 4 °C for 7 days. *SikFBP2* was downregulated in all cold stresses, but its expression level recovered at 0 °C for 24 h. *SikFBP3* was only upregulated at 4 °C for 7 days and *SikFBP6* was upregulated at both 4 °C (7 days) and −2 °C (24 h). Both *SikFBP4* and *SikFBP5* were downregulated in all cold conditions. Regarding the *SikPIPs*, only *SikPIP2*; *7* was upregulated at both 4 °C for 24 h and −2 °C for 12 h. Further, the correlation between RNA-Seq and qPCR was evaluated using log2 expression levels. As shown in Figure 7, the qPCR measurements were moderately correlated with RNA-Seq results (*r* = 0.676, *R*^2^ = 0.444), which indicated that the RNA-Seq data were accurate and could be used for gene expression profile analysis of the cold temperature defense response.

## 3. Materials and Methods

### 3.1. Plant Materials and Stress Treatment

The seedlings of *S. involucrata* were cultured using plant tissue culture technology and placed into a programmable low temperature incubator (Blue R pord, Shanghai-heng scientific instrument company, Shanghai, China) for the cold treatment from 4 to −2 °C in a gradual decrease: 20 °C → 4 °C (24 h) (×2), 20 °C → 4 °C (7 days) (×3), 20 °C → 4 °C (7 days) → 0 °C (24 h) (×4), 20 °C → 4 °C (7 days) → 0 °C (24 h) → −2 °C (24 h) (×5), and directly at −2 °C for 12 h from 20 °C (×6). The whole plant was collected after cold treatment and immediately stored at −70 °C until RNA extraction.

### 3.2. Total RNA Extraction and Sequencing

Total RNA of each sample was isolated using the TRIzol reagent (Invitrogen, Carlsbad, CA, USA) according to the manufacturer’s instructions. The total RNA was sent to the sequencing cooperation of ShengTing for RNA examination and sequencing. HiSeq-2500-Pe of Solexal was selected for the sample transcriptome sequencing.

### 3.3. Transcriptome Assembly and Annotation

The total raw reads were produced by the sequencing machine and the NGSQC v2.3.1 (Aruna Asaf Ali Marg, New Delhi, India) was used to discard these dirty reads to get the clean raw reads. The short read assembly program, Trinity, was used for de novo assembly of the transcriptomes based on the clean paired-end reads [29]. Trinity with min_kmer_cov set to 2 by default and all other parameters also set to default can combine the read according to the overlap of bases to generate a longer sequence, defined contigs. The de novo transcriptome served as the reference transcriptome, and the longest transcript was selected as the unigene to represent one gene.

All unigenes were using BlastX (BLAST+; Bethesda, MD, USA) against with the known public databases, such as NR (NCBI non-redundant protein sequences, *e*-value = 10^−5^), Swissprot (A manually annotated and reviewed protein sequence database, *e*-value = 10^−5^), KEGG (*e*-value = 10^−10^), and COG (*e*-value = 10^−3^). Firstly, the unigene was annotated to define the function of the gene by comparison with the NR, SWISSPROT, and COG databases. Then, through unigene annotation, the GO annotations were based on the results of NR and Pfam using the Blast2GO v2.561 (Available online: www.blast2go.org) with *e*-value = 10^−6^. The TF family was determined by BlastX against the plant TFDB with default *e*-value.

### 3.4. Differential Expression Analysis of Transcripts

The sequence obtained by the Trinity assembly was regarded as the reference genome. All clean reads of each sample were mapped to the reference genome by the RSEM/bowtie program (version 1.2.8; Madison, WI, USA) with default settings. Under the R program package, edge R evaluated the differential expression of unigenes, defined as the RPKM. The cutoff was defined at *p* < 0.0001, BCV = 0.2 and logFC ≥ 2 and FDR < 0.001 to determine the differential expression of genes.

### 3.5. Validation of RNA-Seq Data by qRT-PCR

First-strand cDNA was generated from 3 μg of the same total RNA used for RNA-Seq analysis. The expression level of the target genes was determined by qTR-PCR using the ROCHE LightCycler^®^ 480 system (Salt Lake City, UT, USA) and SYBR Green Real-Time PCR Master Mix (KAPA Biosystems, Wilmington, MA, USA) in 10 μL reactions. Each reaction consisted of 2 ng of total RNA, 0.4 μL of each primer, and 5 μL master mixes. The PCR reactions were performed in a thermocycler with the following conditions: 5 min at 95 °C, 45 cycles of 10 s at 95 °C, 15 s at 60 °C, and 25 s at 72 °C. To verify the presence of a specific product, melting curve analysis of amplification products was performed at the end of each PCR reaction. Primers for qTR-PCR (Table S3) were designed with Primer Premier software (Primer Premier v5.0; Premier Biosoft International, Palo Alto, CA, USA). The comparative *C*_t_ method of quantification was used to quantify the relative expression of specific genes. The *GAPDH* gene was used as the reference. The experiments were repeated three times.

To assess the correlation between different platforms, Pearson’s correlations were calculated using Excel and OriginPro 8.6 (OriginLab; Northampton, MA, USA) to compare the mRNA expression levels measured by RNA-Seq and qRT-PCR (Table S3). To assess treatment effects between different treatment lines, variance analysis was performed using SPSS statistic v17.0 (SPSS Inc.; Chicago, IL, USA).

## 4. Conclusions

*S. involucrata* is an important plant species for studying the response and tolerance of plants to extreme cold conditions. Transcriptional profiling of *S. involucrata* in response to exposure to cold temperatures for different amounts of time was performed using the NGS method. DEGs of the transcriptome under cold stress were identified using NGS technology. Further analysis of these DEGs showed that cold tolerance is a complex process in *S. involucrata*. In *S. involucrate*, the ability to endure cold stress involved many pathways, such as the categories of signal transduction, transcriptional regulation, and water, ion or organic molecules transport, and so on. Some changes were similar to those observed in *Arabidopsis*, rice, *Brachypodium* and *A. mongolicus*. This is the first complete transcriptome resource and the first transcriptome expressional profile generated at different times and under different cold conditions in *S. involucrate*. The results may help our understanding of plant adaptation to harsh environments and *S. involucrata* survival, and provide new insight into the molecular mechanisms of cold and freezing tolerance.

## Figures and Tables

**Table 1:** Summary of *S. involucrata* transcriptome sequencing using the Illumina HiSeq 2500 platform.

Sample	Condition	Clean Reads	Sequence Length (bp)	Total Bases (G)	%GC
×3	20 °C → 4 °C (7 days)	35,597,819	101	3.5	45.56
		35,597,819	101	3.5	45.56
×6	20 °C → −2 °C (12 h)	42,896,402	101	4.3	45.28
		42,896,402	101	4.3	45.28
×5	20 °C → 4 °C (7 days) → 0 °C (24 h) → −2 °C (24 h)	33,720,829	101	3.4	45.45
		33,720,829	101	3.4	45.45
×4	20 °C → 4 °C (7 days) → 0 °C (24 h)	40,779,352	101	4.1	44.73
		40,779,352	101	4.1	44.73
×1	20 °C	38,880,446	101	3.9	47.78
		38,880,446	101	3.9	47.78
×2	20 °C → 4 °C (24 h)	41,863,445	101	4.2	46.32
		41,863,445	101	4.2	46.32
**Assembly Statistic (Transcripts)**					
**Total Sequences**	**Min Length**	**Average Length**	**Max Length**	**N50**	**%GC**
199,758	201	711.37	15,818	1099	39.05

%GC: the percentage of sum of the number of the bases of G and C in the total number of bases.

**Table 2:** Transcripts number involved in signal transduction of *S. involucrata* under cold stress.

Category	4 °C (24 h) ×2		4 °C (7 days) ×3		0 °C (24 h) ×4		−2 °C (24 h) ×5		−2 °C (12 h) ×6	
	Up	Down	Up	Down	Up	Down	Up	Down	Up	Down
Protein kinase	11	15	17	7	11	12	27	23	27	33
Protein phosphatase	9	2	14	1	13	3	22	8	15	5
Receptor-like kinase	12	24	8	41	21	23	32	86	16	42
Histidine kinase	2	0	1	0	1	0	1	1	0	0
Rhythm signal	2	3	1	1	9	1	14	4	2	2
GTP-related	0	1	0	3	0	0	1	7	0	8
Lipid-signal	3	3	3	1	3	4	9	7	0	11
Calcium-related	1	4	0	1	3	6	14	12	0	5

The description of the category of the signal transduction genes. Protein kinase—all the transcripts that possess protein kinase activity but excluding the transcripts of the other categories; Protein phosphatase—the protein having the function phosphoprotein phosphatase activity; Receptor-like kinase—representing receptor-like protein kinase that distribute the plasma membrane; Histidine kinase—protein histidine kinase activity; Rhythm signal—the transcript connected with the pathway of circadian rhythm; GTP-relate—the transcripts with GTPase activity; Lipid-signal—function with phospholipase activity; Calcium-relate—the transcripts related to calcium ion.

**Table 3:** Transcripts number associated with transport under cold temperature in *S. involucrata*.

Category	4 °C (24 h) ×2		4 °C (7 days) ×3		0 °C (24 h) ×4		−2 °C (24 h) ×5		−2 °C (12 h) ×6	
	Up	Down	Up	Down	Up	Down	Up	Down	Up	Down
Ion-related	5	7	5	9	12	5	14	19	12	15
Carbon-related		1	1	5		3	3	2	1	8
Organ-related	3	1	2	3	3		5	8	4	3
ABC family			3	5	5	2	9	8	4	9
Nucleotide	2		1	3	3	1	3	4	4	4
Auxin-related	2	1		3		3	1	3		4
Amino acid	5	3	5	3	5	3	11	7	6	9
Aquaporin	1		1	1	2		2	3		1
MATE efflux family	1		1	3	2	1	5	6	3	
Proton pump	1	1	4		3	2	4	7	3	3
NRT1/PTR family	2	1	3	2	4	3	6	8	4	6
Inorganic salt		2		5			2	7	3	1

The description of the category relative with transporting. Ion-related—the transcripts with ion transporting activity excepting the similar proteins belonged to the other category; Carbon-related—the proteins involved in the carbohydrate transporting; Organ-relate—the proteins with the function of the Niemann-Pick C proteins, Folate transporters, organic cation/carnitine transporters, CMP-sialic acid transporters, lipid-transfer proteins, phosphatidylinositol/phosphatidylcholine transfer proteins, inositol transporters, malate transporters, and phospholipid-transporting ATPases; ABC family—the transcripts that belonged to the ABC super family; Nucleotide—the protein possess the nucleotide transporting activity; Auxin-related—the protein related to the auxin transporting activity; Amino acid—proteins having the amino acid transporting activity; Aquaporin—the water channel proteins; MATE efflux family—the genes that belonged to the MATE efflux family; Proton pump—the relative ATPase; NRT1/PTR family—the genes that belonged to the NRT1/PTR family; Inorganic salt—the proteins that having inorganic salt transporting activity, such as phosphate, nitrate, Ammonium.

## Supplementary Materials

Supplementary materials can be found at www.mdpi.com/1422-0067/18/6/1155/s1.

## Abbreviations

TF	Transcription factor
PE	Paired end
NGS	Next-generation sequencing
qRT-PCR	Quantitative real-time PCR
DEG	Differentially expressed gene
RPKM	Reads per kilo bases per million mapped reads
