# Supplementary figures and images for: De Novo Transcriptome Sequencing and the Hypothetical Cold Response Mode of Saussurea involucrata in Extreme Cold Environments
Source: Int J Mol Sci. 2017 Jun 7;18(6):1155. doi: 10.3390/ijms18061155 (PMC5485979; doi:10.3390/ijms18061155)

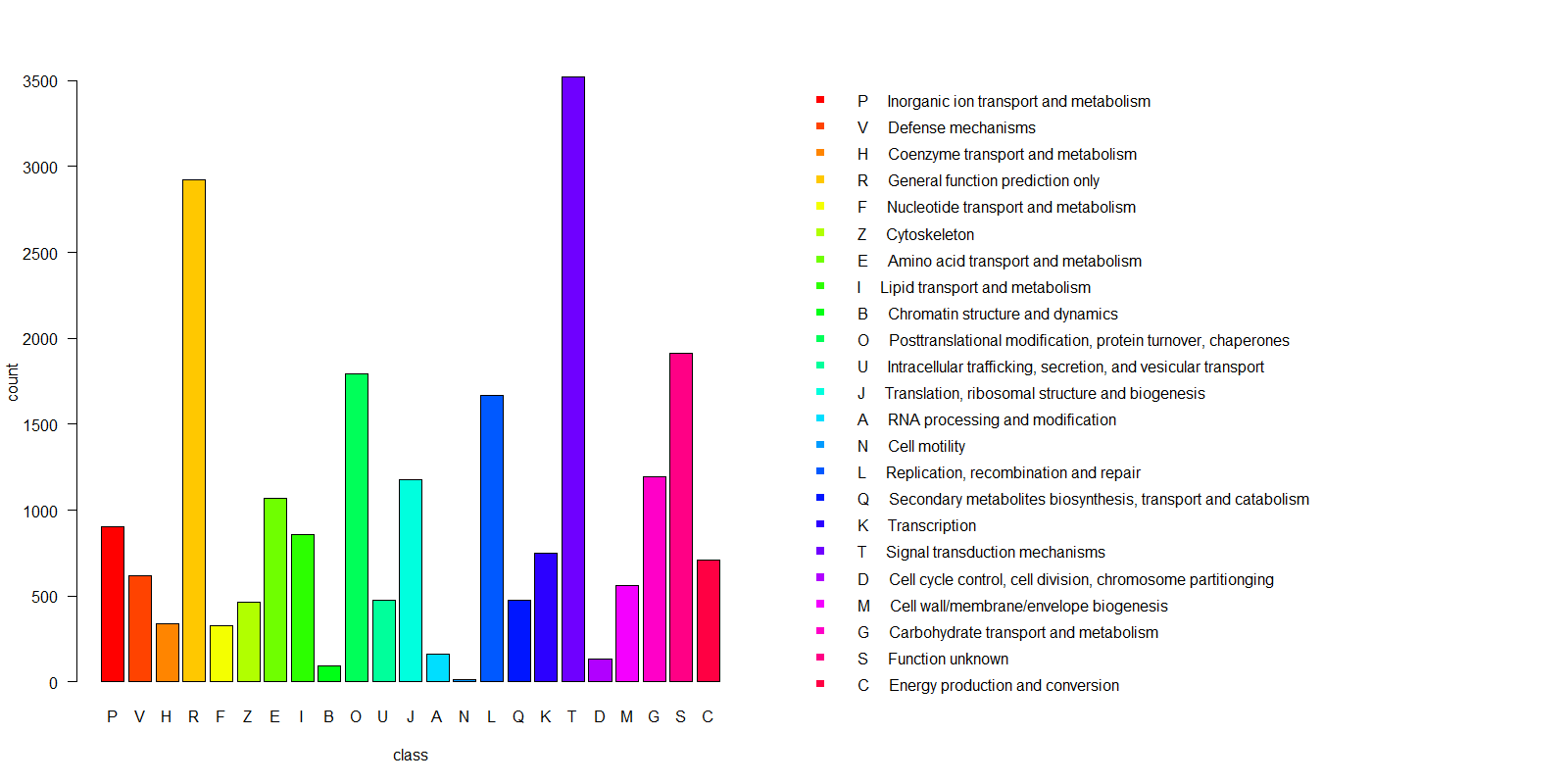

Supplement: Supplementary file 1 [file ijms-18-01155-s001.zip › ijms-188820-supplementary final/Figure S3.tiff]

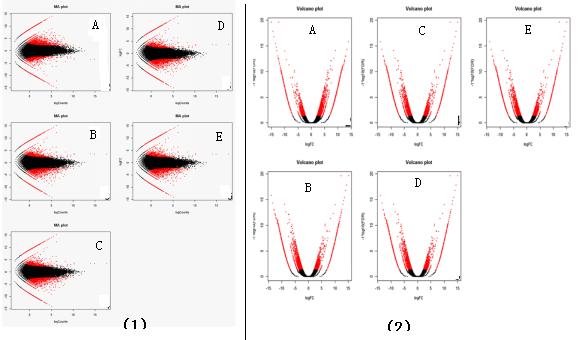

Supplement: Supplementary file 1 [file ijms-18-01155-s001.zip › ijms-188820-supplementary final/Figure S4.png]
